# Nifekalant versus Amiodarone for Out-Of-Hospital Cardiac Arrest with Refractory Shockable Rhythms; a Post Hoc Analysis

**DOI:** 10.22037/aaem.v10i1.1425

**Published:** 2022-01-01

**Authors:** Hiraku Funakoshi, Shotaro Aso, Yosuke Homma, Ryuta Onodera, Yoshio Tahara

**Affiliations:** 1 Department of Emergency and Critical Care Medicine, Tokyobay Urayasu Ichikawa Medical Center, 3-4-32 Todaijima, Urayasu, Chiba 279-0001, Japan.; 2 Department of Clinical Epidemiology and Health Economics, School of Public Health, Graduate School of Medicine, The University of Tokyo. 7-3-1 Hongo, Bunkyo-ku, Tokyo 113-8555, Japan.; 3 Department of Biostatistics & Bioinformatics, Graduate School of Medicine, The University of Tokyo 7-3-1 Hongo, Bunkyo-ku, Tokyo 113-0033, Japan.; 4 Department of Cardiovascular Medicine, National Cerebral and Cardiovascular Center, 6-1 Kishibe-shimmachi, Suita, Osaka 564-8565, Japan.

**Keywords:** Anti-arrhythmia agents, Cardiopulmonary resuscitation, Nifekalant, Ventricular fibrillation, Ventricular flutter

## Abstract

**Introduction:**

It is still unclear that which anti-arrhythmics are adequate for treating refractory dysrhythmia. This study aimed to compare amiodarone and nifekalant in management of out-of-hospital cardiac arrest cases with refractory shockable rhythm.

**Methods:**

This was a post hoc analysis of cases registered in a nationwide, multicentre, prospective registry that includes 288 critical care medical centres in Japan. From June 2014 to December 2017, we included all out-of-hospital cardiac arrest patients aged ≥18 years who presented with refractory arrhythmia (sustained ventricular fibrillation or ventricular tachycardia following delivery of at least two defibrillator shocks) and treated with nifekalant or amiodarone after arrival to hospital. Overlap weight was performed to address potential confounding factors.

**Results:**

1,317 out-of-hospital cardiac arrest patients with refractory arrhythmia were enrolled and categorized into amiodarone (n = 1,275) and nifekalant (n = 42) groups. After overlap weight was performed, there were no significant intergroup differences in increased the rate of admission after return of spontaneous circulation [–5.9% (95%CI: –7.1 to 22.4); p = 0.57], 30-day favourable neurological outcome [0.1% (95%CI: –14 to 13.9); p = 0.99], and 30-day survival [–3.9% (95% CI: –19.8 to 12.0); p = 0.63].

**Conclusion:**

This nationwide study showed that nifekalant was not associated with improved outcomes regarding admission after return of spontaneous circulation, 30-day survival, and 30-day favourable neurological outcome compared with amiodarone.

## 1. Introduction

Approximately 12% of patients with out-of-hospital cardiac arrest (OHCA) survive until discharge from the hospital. In spite of widespread early bystander cardiopulmonary resuscitation (CPR) and automated external defibrillator (AED) use, the overall survival from OHCA remains low and 20% of patients with OHCA who survive develop irreversible neurological disability (1). It was previously reported that only 8% of patients were discharged from the hospital after recovering to a condition in which they could live without any support (1). Thus, the need for a standardized approach to CPR to improve patients’ outcomes after cardiac arrest has been considered for many years. 

For refractory arrhythmia, which is defined as sustained ventricular fibrillation (VF) or pulseless ventricular tachycardia (VT) despite performing defibrillation twice, administration of anti-arrhythmics following administration of adrenaline and defibrillation is recommended, and the guidelines recommend amiodarone administration (2, 3). Although the benefits of administration of anti-arrhythmics are limited, anti-arrhythmics are key for return of spontaneous circulation (ROSC) in cases of refractory arrhythmia (4).

Nifekalant, which was developed and approved in Japan in 1999, is a class III anti-arrhythmic agent per the Vaughan Williams classification. Although a meta-analysis suggested that nifekalant may be effective in improving short-term and long-term survival, the only study that had a low risk of bias among those included in the meta-analysis had shown no benefit from nifekalant. It had also stated that the effect of amiodarone on either of these outcomes could not be confirmed (5). Due to this limitation, nifekalant was an alternative to amiodarone for refractory arrhythmias in the International Liaison Committee on Resuscitation (ILCOR) consensus and nifekalant was not mentioned in the guidelines of the American Heart Association (AHA) and European Resuscitation Council (ERC) (2, 3). In addition, the guidelines released by the Japan Resuscitation Council (JRC) in 2015 recommend the use of amiodarone over nifekalant (6). A large study using a Japanese nationwide in-hospital patient administrative database conducted after the publication of these guidelines compared the effectiveness of nifekalant and amiodarone and found no difference between the two anti-arrhythmics (7). However, the study did not include information on prehospital care, such as bystander witness, bystander CPR, and AED use, which is the most important determinant of the prognoses of patients with cardiorespiratory arrest (8-10). On the other hand, a previous study using prehospital data does not include precise information on in-hospital treatment, and the dose of amiodarone was not consistent in the study. Therefore, evaluation of the effectiveness of nifekalant in the context of data on both pre- and in-hospital care have been the remaining issue. 

Based on the above-mentioned points, this study aimed to compare amiodarone and nifekalant in management of out-of-hospital cardiac arrest cases with refractory shockable rhythm in terms of patients’ admission after ROSC, 30-day favourable neurological outcome (Cerebral Performance Category 1 or 2), and 30-day survival.

## 2. Methods

***2.1 Study design and setting***

This study is a post hoc analysis of cases registered in the Japanese Association for Acute Medicine out-of-hospital cardiac arrest (JAAM-OHCA) registry, which is a nationwide, multi-centre prospective registry that includes 288 critical care medical centres in Japan. The detailed study protocol has been previously described (11). All patients with cardiorespiratory arrest in a prehospital setting who were then transported to a member institution are included in the registry. To allow refusal for inclusion in the registry by patients or their family members, a special committee and each participating institution made available a document regarding opt-out consent on the website and/or a notice board in the emergency department. Therefore, the requirement for informed consent from patients was waived. The registry was approved by the Ethics Committee of Kyoto University (Reference number is R1045), and each hospital also approved the JAAM-OHCA Registry protocol as necessary.

***2.2 Emergency medical system in Japan***

In Japan, all patients with cardiorespiratory arrest are transported by an emergency medical system (EMS) team, because EMS providers are not allowed to terminate resuscitation in the field unless there are obvious signs of death (e.g. lividity or rigor mortis) (12). EMS teams in Japan provide defibrillation via an AED and protect the airway using supraglottic airway or endotracheal intubation and administer adrenaline per remote instruction from a doctor. However, EMS providers are not permitted to administer any anti-arrhythmics.

***2.3 Participants***

This study included all patients in the JAAM-OHCA registry from June 2014 to December 2017. Of these patients, we included all OHCA patients aged ≥18 years in whom refractory arrhythmia was treated with nifekalant or amiodarone. We excluded patients who received both nifekalant and amiodarone.

2.4 Data gathering 

The JAAM-OHCA registry includes both pre- and post-hospitalization data. Prehospitalization data were obtained from the All-Japan Utstein Registry of the Fire and Disaster Management Agency (FDMA) as previously reported (12, 13). In-hospital data were collected via an Internet-based system by physicians or medical staff at each institution. The JAAM-OHCA registry committee integrated the prehospital and in-hospital data, as previously described (12). The protocol was approved by the institutional review board of each participating hospital. During the study period, anonymized data were entered into the web form by the medical staff in charge of the patient and were finally confirmed by the JAAM-OHCA registry committee, which consists of specialists in emergency medicine and epidemiology. A committee member returned any incomplete data forms to the institution submitting the form and the data form was filled out as completely as possible. In-hospital data were systemically combined with prehospital resuscitation data obtained from the All-Japan Utstein Registry of the FDMA, by using the five key items in both datasets: prefecture, emergency call time, age, sex, and cerebral performance category (CPC) 1 month after the OHCA (13).

2.5 Exposure and outcome measures

The exposure was the administration of nifekalant or amiodarone. The responsibility for selecting the anti-arrhythmic medication was entirely entrusted to each physician or institution. Although there are no specific protocols for refractory arrhythmia in the JAAM-OHCA registry, most facilities follow the guidelines published by the JRC, which comply with ILCOR, for performing CPR.

The primary outcome measure of interest was admission after ROSC. The secondary outcomes were 30-day survival and 30-day favourable neurological outcome. A favourable neurological outcome was defined as a CPC score of 1 or 2 (11). The CPC score scale accounts for five outcomes: 1, good cerebral recovery; 2, moderate cerebral disability; 3, severe cerebral disability; 4, coma or vegetative state; and 5, death/brain death (13). 

***2.6 Statistical analysis***

All descriptive statistics were reported as number (%) or mean ± standard deviation. For comparison of characteristic variables among cohorts, independent-sample t-tests were used for numeric variables, while chi-squared tests were used for categorical variables. Overlap weight was performed to address potential confounding (14, 15). A propensity score for nifekalant administration was estimated using a multivariable logistic regression model containing age, sex, witness status, presence of a bystander who performed CPR, aetiology of cardiac arrest (cardiac or not), prehospital adrenaline administration, prehospital airway management, prehospital AED use, response time, physicians, and hospital number. Based on previous studies, these variables would be relevant to treatment assignment (8-10). Overlap weight is defined as 1- propensity score for patients receiving nifekalant and propensity score for patients receiving amiodarone. By applying overlap weights for each patient, we adjusted for the patients’ backgrounds and compared the outcomes.

We used absolute standardized differences to evaluate differences in patient characteristics between the groups. We regarded an absolute standardized difference of <0.1 as an acceptable balancing of covariates between the groups (16). Although data on in-hospital treatments or interventions (veno-arterial extracorporeal membrane oxygenation (VA-ECMO), percutaneous coronary intervention (PCI), and targeted temperature management (TTM)) were collected, these variables were not included in the variables for the estimation of the propensity score, because we assumed that these treatments are administered after ROSC (17). In addition, we also performed a series of sensitivity analyses to examine the robustness of our inference: 1) Based on the assumption that the decision to use these three treatments (VA-ECMO, PCI, and TTM) is made prior to admission, we calculated propensity score including the three treatments and performed an analysis with overlap weight. 2) Analysis was performed excluding patients with ECMO to avoid reverse causation; because it is difficult to identify whether patients with ECMO had resumed spontaneous circulation at the time of admission or not. Stata/SE 16.0 (Stata Corp, College Station, TX, USA) was used for data analyses. The threshold for significance was set at P < 0.05.

## 3. Results

***31 Baseline characteristics***

**Figure 1:**
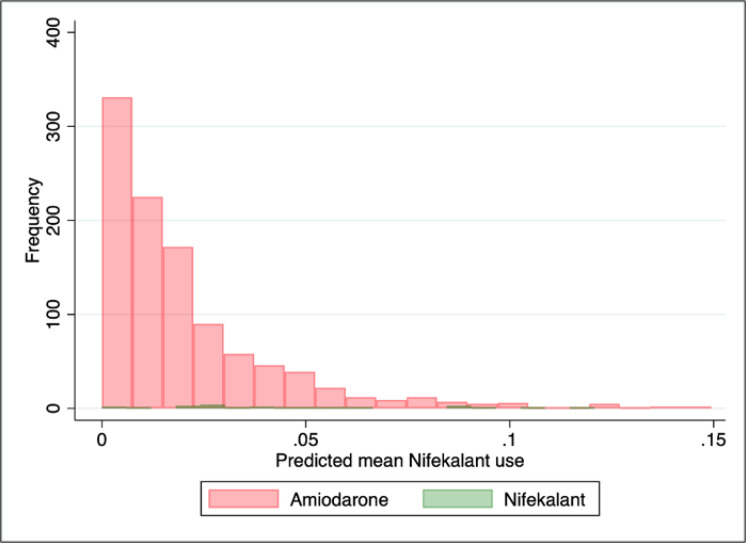
The histogram of propensity score distribution in each treatment group

During the study period, 1,374 OHCA patients with refractory arrhythmia were enrolled in this study. Of these patients, 1,317 eligible patients were included in this analysis after excluding 57 patients who had received both nifekalant and amiodarone. These patients were categorized into amiodarone (n = 1,275) and nifekalant (n = 42) groups. Table 1 compares the baseline characteristics of studied cases between nifekalant and amiodarone groups. The mean patients’ age was 64.0 ± 15.4 years (20.7% female). Propensity score based on the variables listed in methods section are presented in figure 1. After overlap weight adjustment for treatment with nifekalant, baseline patient characteristics were well-balanced between the two groups, as shown in table 2 (p < 0.01). 

***3.2 Comparing outcomes***

In crude data, the proportions of admission after ROSC in nifekalant group were not significantly different from those in amiodarone group (64.3% vs. 56.6%, p = 0.32). The proportions of the favourable 30-day neurological outcome were significantly higher among patients who received nifekalant compared to patients for whom amiodarone was administered (23.8% vs. 10.4%, p < 0.01). The proportions of 30-day survival in nifekalant group were not significantly different from those in amiodarone group (31.0% vs 20.1%, p = 0.09). With overlap weight, nifekalant use was not associated with improved outcomes (Hospital admission after ROSC: risk difference, –5.9% (95% CI: -26.7 to 14.8); favourable 30-day neurological outcome: risk difference, 0.1% (95% CI: –14 to 13.9); 30-day survival: risk difference, –3.9% (95% CI: –19.8 to 12.0) (Table 3)). 

***3.3 Key in-hospital treatments***

Table 4 shows differences in key in-hospital treatments or interventions between the two groups. The proportions of patients who received veno-arterial extracorporeal membrane oxygenation (VA-ECMO), percutaneous coronary intervention (PCI), and target temperature management (TTM) were higher in the nifekalant group. Intergroup differences in these proportions were not significant. Although the proportions of the patients who received VA-ECMO were well-balanced after overlap weight, the proportions of those who received the percutaneous coronary intervention and targeted temperature management remained different between the two groups (Table 4). 

In the sensitivity analysis, VA-ECMO, PCI, and TTM were well-balanced between the two groups with overlap weight using another propensity score, and nifekalant use showed no significant association with higher proportion of 30-day favorable neurological outcome [0.3% (95% CI: –14.2 to 13.6)] and 30-day survival [–4.6% (95% CI: –20.6 to 11.3)]. As with the results of main analysis, after excluding patients with ECMO, nifekalant use showed no significant association with higher proportion of admission after ROSC [risk difference: –0.4% (95% CI: –20.6 to 13.0)].

## 4. Discussion

In this nationwide study, we compared the proportions of admission after ROSC and 30-day outcomes between patients treated with nifekalant and those treated with amiodarone after OHCA associated with refractory arrhythmias. After adjusting for patients' background, including both pre-hospital and in-hospital data by overlap weight, no significant difference in the proportions of admission after ROSC and 30-day favourable outcomes was found between nifekalant and amiodarone administration after arrival in hospital. 

This study compared the effectiveness of nifekalant with that of amiodarone by using data including detailed prehospital care data from a nationwide registry of patients with cardiorespiratory arrest. Amiodarone and nifekalant are both classified as class III antiarrhythmic agents (potassium channel blockers) in the Vaughan‐Williams classification. However, these two antiarrhythmic drugs have different pharmacological characteristics (18, 19). Amiodarone is not only a potassium channel blocker but also has blocking effects on other ion channels such as sodium and calcium channels and α and β receptors. As a result of these multi-channel blocking effects, amiodarone has negative inotropic and vasodilatory effects, which may in turn negatively affect the haemodynamic status for coronary artery flow and after ROSC in OHCA patients. On the other hand, nifekalant is a pure potassium channel blocker that does not block sodium or calcium channels (20). In a study conducted using an animal model, nifekalant use decreased the defibrillation threshold for ventricular fibrillation (21). From these pharmacological points of view, nifekalant could have advantages over amiodarone for the treatment of refractory arrhythmia. However, clinical studies are limited. Amino et al. reported that nifekalant and amiodarone were both associated with improved 24-hour survival compared to lidocaine in OHCA patients with refractory arrhythmia (22). However, this study did not perform a direct comparison between nifekalant and amiodarone. In addition, because the dose of amiodarone was not standardized during the study period, only approximately 200 people received the international standard dose of 300 mg (22).

In this study, only 3.2% (57/1374) of the patients received nifekalant. Because nifekalant was recommended alongside amiodarone for refractory arrhythmias until 2005, the use of nifekalant was relatively common in previous studies (7, 22). The removal of nifekalant from the recommendations in the 2015 guideline of the AHA could result in a decrease in the use of nifekalant (2). In the 2020 guideline of the AHA, the use of nifekalant for refractory arrhythmias has not been included. Thus, Japan is a rare country in which nifekalant was included as an alternative to amiodarone to treat OHCA patients with refractory arrhythmia. High-quality studies with sufficient size are warranted to determine which antiarrhythmic drugs are effective for the treatment of refractory arrhythmias with greater confidence across the country.

**Table 1:** Comparing the baseline characteristics of studied cases between amiodarone (n = 1,275) and nifekalant (n = 42) groups

**Variables**	**Total**	**Amiodarone**	**Nifekalant**	**SD**	**P **
**Age (year)**	64.0 ± 15.4	64.1 ±15.4	60.1±16.3	0.25	0.14
**Gender (female) **	273 (20.7)	267 (20.9)	6 (14.3)	0.18	0.30
**Witness**	929 (70.5)	900 (70.6)	29 (69.0)	0.03	0.83
**Bystander CPR**	581 (44.1)	564 (44.2)	17 (40.5)	0.08	0.63
**AED use**	933 (74.4)	897 (70.4)	36 (85.7)	0.42	0.02
**Prehospital adrenaline **	495 (37.6)	485 (38.1)	10 (23.8)	0.31	0.06
**Airway equipment use**	656 (59.3)	645 (50.6)	11 (26.2)	0.24	0.26
**Physician on scene**	280 (21.3)	273 (21.4)	7 (16.7)	0.12	0.46
**Presumed ** **cause of CPA**	1,138(86.4)	1,098(86.1)	40 (95.2)	0.32	0.09
**Call to dispatch (minute)**	8.5 ±3.5	8.6 ± 3.5	7.8 ± 2.4	0.24	0.15

Data are presented as mean ± standard deviation or number (%). Abbreviations: AED, automated external defibrillator; CPA, cardiopulmonary arrest; CPR, cardiopulmonary resuscitation; SD, Standardized difference.

**Table 2:** Overlap propensity score-weighted characteristics in amiodarone (n = 1,275) and nifekalant (n = 42) groups

**Variables**	**Amiodarone**	**Nifekalant**	**SD**
Age (year)	61.9 ±15.9	61.9 ± 17.5	<0.01
Female sex	9.0	9.0	<0.01
Witness	73.8	73.8	<0.01
Bystander CPR	43.8	43.8	<0.01
AED use	82.4	82.4	<0.01
Prehospital adrenaline	26.6	26.6	<0.01
Airway equipment use	48.7	48.7	<0.01
Physician on scene	9.1	9.1	<0.01
Presumed cause of CPA	91.0	91.0	<0.01
Call to dispatch (minute)	7.72 ± 2.53	7.72 ± 2.43	<0.01

Data are presented as mean ± standard deviation or number (%). Abbreviations: AED, automated external defibrillator; CPA, cardiopulmonary arrest; CPR, cardiopulmonary resuscitation; SD, standardized difference.

**Table 3:** Comparisons of outcomes between amiodarone (n = 1,275) and nifekalant (n = 42) groups

**Variables**	**Amiodarone**	**Nifekalant**	**RD (95% CI)**
**Unadjusted analysis**			
Hospital admission after ROSC	56.6	64.3	7.6 (–7.1 - 22.4)
30-day favourable neurological outcome	10.4	23.8	13.5 (0.46 - 26.5)
30-day survival	20.1	31.0	10.9 (–3.3 - 25.0)
**Weighted analysis**			
Hospital admission after ROSC	62.1	56.2	–5.9 (–26.7 -14.8)
30-day favourable neurological outcome	12.8	12.8	0.1 (–14.0 - 13.9)
30-day survival	21.2	17.3	–3.9 (–19.8 -12.0)

Data are presented as percentage. Abbreviations: ROSC, return of spontaneous circulation; RD: risk difference; CI: confidence interval.

**Table 4:** Comparing the treatments and interventions after return of spontaneous circulation between amiodarone (n = 1275) and nifekalant (n = 42) groups

**Treatment**	**Un-weighted**				**Weighted**		
	**Amiodarone**	**Nifekalant**	**SD**	**P **	**Amiodarone**	**Nifekalant**	**SD**
**VA-** **ECMO**	40.5	50.0	0.19	0.22	46.4	47.2	0.02
**PCI**	23.5	31.0	0.17	0.27	27.2	21.6	-0.13
**TTM**	29.1	47.6	0.38	<0.01	32.4	38.5	0.13

Data are expressed as %. PCI, Percutaneous coronary intervention; TTM, Target temperature management; VA-ECMO, veno-arterial extracorporeal membrane oxygenation. SD: standardized difference.

## 5. Limitations

There are several limitations in this study. First, as with any observational study, there was a risk of selection bias. Especially in this study, the number of patients in nifekalant group was limited, which may lead to differences in patient distribution. As a result, comparability may be compromised. In addition, there was a risk of confounders. To overcome this limitation, we performed overlap weight, and all measured confounding factors were well balanced among each group, yet the risk of unmeasured confounder remained. Second, the power of this study was low due to the small number of patients in the nifekalant group. However, it has been pointed out that it is inappropriate to judge the appropriateness of a study based on its post hoc power, and the results should be interpreted with caution (25). Third, we could not adjust for treatment and intervention after ROSC and during admission. As described in the results, the proportions of patients who received target temperature management and percutaneous coronary intervention differed between the nifekalant and amiodarone groups. However, even in randomized controlled trials, statistical adjustment for factors after group allocation could introduce over-adjustment bias and could inappropriately dilute the true relationship between the exposure and outcome in the study (17). Therefore, we conducted a sensitivity analysis using propensity score including the three treatments and performed an analysis with overlap weight based on the assumption that the decision to use these three treatments (VA-ECMO, PCI, and TTM) is made prior to admission. The result is consistent, which may support the robustness of the results. Fourth, although dose and timing of anti-arrhythmics is an important factor, JAAM-OHCA registry does not include the time of administration or the dose of anti-arrhythmics. 

## 6. Conclusion

This nationwide study showed that nifekalant was not associated with improved outcomes regarding admission after return of spontaneous circulation, 30-day survival, and 30-day favourable neurological outcome compared with amiodarone.

## 7. List of Abbreviations

AED, Automated external defibrillator; AHA, American Heart Association; CPC, Cerebral performance category; CPR, Cardiopulmonary resuscitation; EMS; Emergency medical system; ERC, European Resuscitation Council; FDMA, The Fire and Disaster Management Agency; ILCOR, International Liaison Committee on Resuscitation; JAAM-OHCA, The Japanese Association for Acute Medicine out-of-hospital cardiac arrest; JRC, Japan Resuscitation Council; OHCA, Out-of-hospital cardiorespiratory arrest; ROSC, Return of spontaneous circulation; VF, Ventricular fibrillation; VT, Ventricular tachycardia

## 8. Declarations

### 8.1 Acknowledgement

None.

### 8.2 Funding

The authors received no financial support for the research, authorship and/or publication of this article.

### 8.3 Authors’ contributions

HF and SA performed the quantitative analysis and drafted the manuscript, which was critically reviewed by all of the authors. HF, YH, RO and YT planned the study and analyzed and interpreted the data. All authors read and approved the final manuscript.

### 8.4 Conflict of interest

The authors declare that they have no competing interests.

### 8.5 Ethical consideration

The study was conducted according to the ethical guidelines of the Declaration of Helsinki. The patients’ information was anonymized and de-identified prior to the analysis, thus the informed consent of the patient was waived.
